# Socioeconomic and geographic inequalities in unmet healthcare needs in Cambodia: evidence from a national cross-sectional study

**DOI:** 10.1186/s12939-026-02922-y

**Published:** 2026-06-18

**Authors:** Khin Thiri Maung, Monica Hunsberger, Heng Sopheab, Nawi Ng, Ailiana Santosa

**Affiliations:** 1 School of Public Health and Community Medicine, Institute of Medicine, Sahlgrenska Academy, University of Gothenburg, Gothenburg, Sweden; 2 School of Public Health, National Institute of Public Health, Phnom Penh, Cambodia

**Keywords:** Health inequalities, Unmet healthcare needs, Cambodia

## Abstract

**Introduction:**

Despite economic growth and health system improvements in Cambodia, inequalities in access to healthcare services persist. Unmet healthcare needs provide a key indicator of equity in health service access. This study examined the association between socioeconomic position (SEP) and unmet healthcare needs among Cambodian adults and assessed whether these associations differed by residential area.

**Method:**

We conducted a cross-sectional analysis of nationally representative data from 5,001 adults aged ≥ 18 years in Cambodia who participated in the 2023 World Health Survey Plus (WHS+). Unmet healthcare need was defined using three specifications: (1) not receiving healthcare despite reported need; (2) not receiving healthcare or receiving care from a non-medical facility; and (3) not receiving healthcare or receiving care from a non-medical facility or private pharmacy. SEP was derived using principal component analysis conducted separately for urban and rural households and categorized into tertiles. Survey-weighted multivariable logistic regression examined associations between SEP and unmet healthcare needs stratified by residential area.

**Results:**

The prevalence of unmet healthcare needs ranged from 7.6% to 42.8%, depending on the definition used. In urban areas, no statistically significant associations between SEP and unmet healthcare needs were observed across specifications. In rural areas, low-SEP individuals had significantly higher odds of unmet healthcare needs under Specification 2 (aOR = 2.40, 95% CI: 1.06–5.41), while middle-SEP individuals had lower odds under Specification 3 (aOR = 0.63, 95% CI: 0.41–0.96). Predicted probabilities varied across SEP and residential area, with patterns differing depending on outcome specifications, particularly in the urban area.

**Conclusion:**

Unmet healthcare needs in Cambodia show persistent socioeconomic and geographic inequalities, which are more evident when defined by access to formal providers. Self-reported measures may underestimate these disparities. Policies should prioritize strengthening affordable rural primary care, regulating private pharmacies, and improving financial protection for disadvantaged populations.

**Supplementary Information:**

The online version contains supplementary material available at 10.1186/s12939-026-02922-y.

## Background

Cambodia, with an estimated population of 17.6 million in 2024, [1] has experienced substantial economic growth and improvements in population health over the past two decades [2]. Life expectancy at birth increased from approximately 58.2 years in 2000 to 70.7 years in 2024. However, these gains have not been matched by comparable improvements in health-adjusted life expectancy, suggesting that additional years of life are not consistently lived in good health [3, 4]. This gap highlights ongoing challenges related to quality of life, timely access to care, and the management of chronic conditions. Despite overall progress, significant socioeconomic and geographic disparities in access to essential healthcare services persist [5]. Individuals in rural areas and low-income households continue to face structural barriers, including limited service availability, financial constraints, and geographic isolation [6]. These barriers contribute to unequal access to needed care and may reinforce broader social and health inequalities [7].

Achieving Universal Health Coverage (UHC), ensuring that all individuals receive needed care without financial hardship, remains a key national priority. From an equity perspective, monitoring UHC requires not only assessing service coverage but also identifying populations whose healthcare needs remain unmet [8]. Self-reported unmet healthcare needs, defined as the perception of not receiving necessary medical care, are widely used as an indicator of health system performance and equity, particularly in low- and middle-income countries [9–12]. However, it is a multidimensional construct, shaped not only by structural barriers such as cost and distance, but also by care-seeking behavior, and the types of providers individuals use [13].

In many low-resource settings, including Cambodia, pharmacies and non-medical providers often serve as the first point of contact for healthcare, despite concerns about the quality of care provided [14–16]. In addition, many medicines, including antibiotics and other prescription medications, are frequently available without a prescription in these settings [17, 18]. Pharmacies may therefore provide not only medication dispensing but also basic symptom assessment, treatment advice, and referral guidance, and are widely used for common acute illnesses and chronic disease management [19]. However, pharmacy-based care may also substitute for more comprehensive formal healthcare services when access to diagnostic evaluation, continuity of care, or physician-led treatment is limited [20]. In LMICs, a substantial proportion of pharmacies are operated by non-pharmacist personnel or diploma-level providers with authority to dispense medicines [21]. Differences in provider qualifications, regulation, and dispensing practices may affect the quality and appropriateness of care and may also create uncertainty among patients regarding professional standards of care [21]. Consequently, while pharmacy utilization may improve physical access to healthcare, it may also mask unmet healthcare needs when conditions require formal diagnosis, continuity of care, or comprehensive treatment [22–24]. Estimates of unmet healthcare need may therefore vary depending on whether care obtained from pharmacies and other informal providers is considered as meeting healthcare need or as indicative of unmet healthcare need.

Most existing studies typically rely on a single definition of unmet healthcare need based on respondents’ subjective assessment if their needs are met, irrespective of the quality of care provided, [25, 26] which may obscure important differences in unmet healthcare needs and lead to inconsistent findings across studies. A more nuanced approach that distinguishes between lack of any care, reliance on informal providers, and pharmacy-based care may provide a more comprehensive understanding of healthcare access and equity. However, empirical evidence comparing these alternative definitions remains limited.

This study is guided by Andersen’s Behavioral Model of Healthcare Utilization [27] and the Social Determinants of Health framework [28], which conceptualize healthcare access as the result of interactions between predisposing, enabling, and need factors. Within this framework, socioeconomic position (SEP) and geographic location are key enabling determinants that shape individuals’ ability to obtain appropriate care. SEP reflects access to material and social resources, while geographic location captures differences in service availability and spatial accessibility. Together, SEP and geographic location may generate compounded disadvantage, particularly among rural populations with limited financial resources [29]. Using nationally representative data from Cambodia, this study examines the association between SEP and unmet healthcare needs among Cambodian adults and assesses whether these associations differ by residential area. By applying multiple provider-based definitions of unmet healthcare need, this study aims to provide a more nuanced assessment of healthcare access and generate equity-relevant evidence to inform health system planning and targeted policy interventions.

## Materials and methods

### Study design, data source and study population

**Fig. 1:**
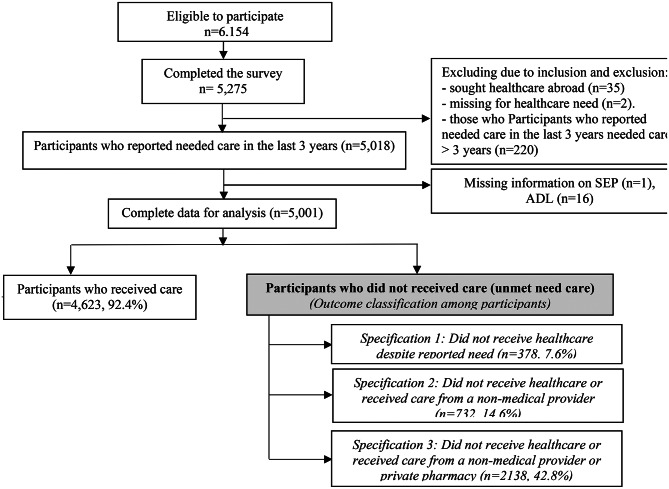
Flow diagram for participants’ healthcare access and distribution of unmet healthcare needs among Cambodian adults, WHS+ Cambodia 2023

This cross-sectional study used nationally representative data from the World Health Survey Plus (WHS+), conducted by the National Institute of Public Health in Cambodia between March 12 and May 31, 2023, among adults aged 18 years and older. The WHS + is a World Health Organization (WHO) led global study designed to provide population-level evidence on health status, healthcare access, and health system performance. A three-stage cluster sampling design was applied. In the first stage, 276 villages were selected from a total of 14,568 villages based on 2021 household and population data provided by the National Institute of Statistics (NIS) using probability proportional to size (PPS). This approach ensured proportional representation of villages by population size across the country. In the second stage, 22 households were randomly selected per village. High-resolution satellite images and GIS software were used to identify all buildings in each selected village, and geographic coordinates were mapped in Google Earth to generate a line-listed sampling frame. A total of 44 structures per village were initially selected based on pre-test findings, which indicated that approximately half would meet the eligibility criteria. Eligible households were defined as private residences with at least one permanent resident aged 18 years or older who had lived in the village for six months or more in the previous year. This residency criterion was applied to ensure stable exposure to local healthcare contexts. In the third stage, one household head completed the household questionnaire, and one adult was randomly selected to complete the individual questionnaire. Respondent selection was performed using a computer-assisted personal interviewing (CAPI) system. If the selected individual was unavailable or refused to participate, no replacement was made, thereby preserving the probabilistic sampling design. Of 6,154 eligible participants, 5,275 completed the survey, corresponding to a response rate of 85.7%. For the present analysis, the analytical sample was restricted to participants who reported needing healthcare within the past three years, based on responses to “When was the last time you needed healthcare?”. Consistent with the study definition and frameworks from the Organization for Economic Co-operation and Development (OECD), unmet healthcare was defined based on whether care was received for the reported need within the same timeframe.^30^ Participants who did not report a need for healthcare during this period were not included in the analysis, as unmet healthcare need could not be assessed (*n* = 220, 4.2%). In addition, participants were excluded if they reported seeking healthcare abroad (*n* = 35, 0.67% of the respondents), as their healthcare experiences were not comparable to the domestic healthcare system, or if they had missing or incomplete responses for the healthcare utilization module (*n* = 2, 0.04%), had missing information on SEP (*n* = 1, 0.02%), or had missing data on activities of daily living (ADL) (*n* = 16, 0.32%). After applying these inclusion and exclusion criteria, the final analytical sample comprised of 5,001 Cambodian adults (Fig. 1).

### Outcomes

The primary outcome was unmet healthcare needs. Healthcare access and utilization were assessed using two questions capturing (i) timing of the most recent need of healthcare and (ii) whether care was received for that need within the past three years. Participants who did not receive healthcare despite reporting a healthcare need within the past three years were classified as having unmet healthcare needs (*Specification 1*).

Among participants with met needs, we further classified the type of facility most often used for care based self-reported healthcare seeking behavior on responses to the question: “Where did you go most often when you felt sick or needed to consult someone about your health?” Responses were grouped into three categories:

1. Public facilities – national hospitals, referral hospitals, health centers, and health posts.
2. Private facilities – private hospitals or clinics, pharmacies, or home/office visits by trained healthcare workers.
3. Non-medical facilities – unqualified drug shops, traditional healers, traditional birth attendants, and magicians.

To assess the robustness of the findings, we constructed two additional definitions of unmet healthcare needs. In *Specification 2*, participants were classified as having unmet healthcare need if they either did not receive healthcare or if they received care from a non-medical facility. In *Specification 3*, unmet healthcare needs additionally included care obtained from a private pharmacy and were defined as either not receiving healthcare or receiving care from a non-medical facility or private pharmacy.

Given the central role of community pharmacies as first-contact healthcare providers in many LMICs, including Cambodia, pharmacy utilization does not necessarily indicate inappropriate care [19]. However, reliance on pharmacies may reflect substitution for formal diagnostic and treatment services in contexts were financial, geographical, or structural barriers limit access to comprehensive healthcare [31]. Therefore, Specifications 2 and 3 were conceptualized as sensitivity definitions intended to capture potential unmet need for formal healthcare services, rather than to classify pharmacy-based care as inherently inadequate.

The frequency and weighted proportion of participants reporting healthcare access, as well as the distribution of unmet healthcare needs across the three specifications, were presented in Supplementary Table S1.

### Exposure

Housing and asset variables were used to construct a household wealth index following the Demographic and Health Survey (DHS) methodology [32]. Only variables with sufficient variation in ownership (9% − 90%) were retained for analysis and assessed using the Keiser-Meyer-Olkin test. Principal Component Analysis (PCA) was applied to the correlation matrix of these variables to derive SEP, which was operationalized as a relative measure reflecting households’ position within the socioeconomic hierarchy rather than an absolute socioeconomic measure. Given that patterns of asset ownership and housing conditions differ between rural and urban settings, PCA was conducted separately for urban and rural households using different indicators (see Supplemental Table S2). This approach was intended to better capture relative SEPs within each setting and to avoid potential SEP misclassification, particularly by disproportionately classifying rural households into lower SEP categories that may arise from applying a pooled PCA. The first principal component, which explained the largest proportion of variance (27.7% in rural areas and 30.1% in urban areas), was used to construct the wealth index. The resulting wealth scores were ranked in ascending order and divided into tertiles. Tertiles were used instead of quintiles to ensure sufficient sample size and stable estimates within each category, especially for analyses examining the combined associations of SEP and residential area. To facilitate analysis, the urban- and rural- specific SEP indices were subsequently combined into a single categorical variable (low, middle, high), reflecting SEP within each residential context.

### Covariates

Covariate selection was guided by a conceptual framework based on Andersen’s Behavioral Model of Healthcare Utilization, which considers predisposing (e.g., age, sex, education), enabling (e.g., SEP, residence), and need factors (e.g., chronic conditions, functional status). Variables were included as potential confounders based on their theoretical relationships with both the exposure (SEP) and the outcome (unmet healthcare need).

***Sociodemographic factors*** included age groups (18–49, 50–59, and ≥ 60 years), sex (male, female), marital status (currently married; never married/divorced/widowed), and education level (no or incomplete primary education, completed primary education, completed secondary education, and higher).

***Residential area*** was classified as urban or rural using geographic dwelling information available as part of the sampling frame. This variable was included because urban populations generally have better access to water and sanitation [33] and are more likely to receive priority in infrastructure development [34, 35].

***Chronic conditions*** were assessed using self-reported physician diagnoses of eight chronic diseases within the preceding 12 months: arthritis, stroke, angina, diabetes, chronic lung disease, asthma, depression, and hypertension. Participants were categorized as having no chronic condition, one chronic condition, or multimorbidity (two or more chronic conditions) [36].

***Functional status*** was evaluated using six Activities of Daily Living *(ADL)*: bathing, dressing, toileting, transferring (getting in and out of bed or chair), eating, and maintaining continence, based on the Katz Index of Independence in Activities of Daily Living and related functional assessment frameworks [37]. We used the threshold of reporting any difficulty in at least one ADL domain to capture the full spectrum of functional limitations that may affect healthcare access and utilization. Restricting the definition to more severe limitations would likely underestimate the burden of functional difficulties and exclude individuals who may already experience barriers to obtaining healthcare. For each task, participants reported their level of difficulty over the past 30 days on a Likert scale ranging from “no difficulty” to “cannot do/extreme difficulty”. Participants were classified as having functional limitation if they reported any level of difficulty in performing at least one ADL [38, 39].

### Statistical analyses

The proportion of missing data across study variables ranged from 0.02% to 0.32%, with the highest level of missingness observed in the covariate functional status variable (0.32%). Due to the minimal level of missingness, only complete cases were included in the analysis (*n* = 5001).

All analyses incorporated survey weights provided in the original survey dataset to ensure representativeness of the adult population in Cambodia. Regression analyses further account for the complex survey design by incorporating clustering at the primary sampling unit (village) level and stratification variables specified in the survey design. Survey-specific commands in Stata (*svy* procedures) were used to obtain appropriate standard errors and confidence intervals.

Descriptive statistics were used to summarize respondents’ sociodemographic, socioeconomic, and health-related characteristics as weighted proportions. Differences between those with and without unmet healthcare needs were examined across the three outcome specifications. Associations between SEP, residential area, and unmet healthcare needs across Specifications 1–3 were examined using multivariable logistic regression models. Logistic regression was selected as the outcomes were binary and the objective was to estimate adjusted associations between exposures and unmet healthcare needs. Models were adjusted for age, sex, marital status, education, residential area, chronic conditions, and functional status, selected a prior based on their established associations with both SEP and healthcare access.

We assessed key model assumptions, including the appropriateness of logistic regression for binary outcomes, independence of observations within the survey design, and absence of problematic multicollinearity. Multicollinearity among independent variables was assessed using variance inflation factors (VIFs). All VIF values were below 2, with a maximum VIF of 1.59, indicating no evidence of problematic multicollinearity among the covariates included in the adjusted models.

To examine whether the association between SEP and unmet healthcare need differed by residential area, we conducted stratified analyses by presenting results separately for urban and rural populations. This approach was chosen because SEP was constructed using separate PCA within each residential stratum, reflecting relative SEP within each context rather than a directly comparable absolute scale across the full sample. A pooled interaction term would therefore be difficult to interpret. Within each stratum, high SEP was used as the reference category. Predicted probabilities of unmet healthcare need across SEP groups and residential areas were estimated using marginal standardization and presented graphically to illustrate the pattern of socioeconomic gradients within each residential stratum.

Results are presented as adjusted odds ratios (aOR) with 95% confidence intervals (CI). All analyses were conducted using Stata 18.0 (StataCorp LLC, College Station, TX, USA).

### Ethical considerations

Ethical approval for the WHS+ Cambodia was obtained from the National Ethics Committee for Health Research, Kingdom of Cambodia (No.221-NECHR). Verbal informed consent was obtained from all participants before data collection by the trained interviewer. The original study protocol initially specified written informed, however, following respondents’ concerns regarding the potential misuse of signatures on written consent forms, approval was obtained from the NECHR to implement verbal consent for this survey. This study was exempted from review by the Swedish Ethical Review Authority (Dnr-2023-02807-01).

## Results

As shown in Fig. 1, among 5,001 participants who reported needing healthcare within the past three years, 4,623 participants (92.4%) reported receiving care, while 378 (7.6%) reported unmet healthcare needs under Specification 1. When broader definitions were applied, the proportion classified as having unmet healthcare needs increased 14.6% (*n* = 732) under Specification 2 and to 42.8% (*n* = 2,138) under Specification 3, which included non-medical facilities and private pharmacies.

Table 1 summarizes participant characteristics across the three specifications of unmet healthcare needs. Under *Specification 1*, unmet healthcare needs were more frequently reported among adults aged 18–49 years (8.6%), men (8.4%), no chronic condition (8.6%), and individuals in the lowest SEP (9.4%). Under *Specification 2*, unmet healthcare needs were higher and most common among adults in the lowest SEP (17.7%). In contrast, under *Specification 3*, unmet healthcare needs were more prevalent among urban residents (48.6%) and no chronic conditions (45.4%)

**Table 1:** Frequency and weighted proportion of unmet healthcare needs across three outcome specifications among Cambodian adults, WHS+ Cambodia 2023

Characteristics	Specification 1 (*n* = 378)		Specification 2 (*n* = 732)		Specification 3 (*n* = 2138)	
	*n*	Weighted (%)	*n*	Weighted (%)	*n*	Weighted (%)
**Socioeconomic Position**						
Low	156	9.4	294	17.7	714	42.9
Middle	114	6.8	249	14.8	707	41.9
High	108	6.5	189	11.5	717	43.5
**Residential area**						
Urban	138	7.0	240	12.2	953	48.6
Rural	240	7.9	492	16.2	1185	39.0
**Sex**						
Men	126	8.4	230	15.3	727	48.2
Women	252	7.2	502	14.4	1411	40.4
**Age group (in year)**						
18–49	232	8.6	407	15.0	1163	43.0
50–59	67	6.5	155	14.9	451	43.5
≥ 60	79	6.3	170	13.5	524	41.7
**Marital status**						
Married	307	7.9	563	14.6	1508	38.9
Divorced/Widowed/Never Married	71	6.3	169	14.9	469	41.2
**Education level**						
No education/Incomplete primary	222	7.8	467	16.4	1212	42.7
Complete primary	80	7.0	138	12.1	484	42.4
Complete at least secondary	76	7.4	127	12.4	442	43.3
**Presence of chronic conditions**						
None	253	8.6	488	16.5	1338	45.4
One	85	6.4	163	12.3	541	40.9
Two or more	40	5.5	81	11.1	259	35.6
**Functional status**						
No difficulty	153	7.4	296	14.3	897	43.5
With difficulty	225	7.7	436	14.8	1241	42.2

Footnote: n= frequency, % = weighted row percentage. **Specification 1**: Did not receive healthcare despite reporting a healthcare need within the past three years, **Specification 2**: Did not receive healthcare or received care from a non-medical facility, and **Specification 3**: Did not receive healthcare or received care from a non-medical facility or private pharmacy

### Socioeconomic position and unmet healthcare needs stratified by residential area across three outcome specifications

Table 2 presents the stratified associations between SEP and unmet healthcare needs by residential area across the three outcome specifications. In urban areas, no statistically significant associations were observed between SEP and unmet healthcare needs across all three specifications. However, low- and middle-SEP groups consistently showed higher odds compared with the high-SEP reference group in Specifications 1 and 2, while estimates in Specification 3 suggested little difference between SEP groups. In rural areas, associations between SEP and unmet healthcare needs were more pronounced and varied across specifications. No statistically significant association were observed under Specification1. In Specification 2, low-SEP rural residents had significantly higher odds of unmet healthcare needs compared with high-SEP rural residents (aOR = 2.40, 95% CI: 1.06–5.41). In Specification 3, low-SEP showed lower odds of unmet healthcare needs compared with high-SEP rural residents, although the association was not statistically significant. In contrast, middle-SEP rural residents had significantly lower odds of unmet healthcare needs (aOR = 0.63, 95% CI: 0.41–0.96). The full adjusted models examining the associations between SEP and unmet healthcare needs among urban and rural populations are presented in Supplementary Tables S3 and S4

**Table 2:** Stratified association between socioeconomic position and unmet healthcare needs by residential area among Cambodian Adults, WHS+ Cambodia 2023

Socioeconomic position by residential area	Specification 1 aOR (95% CI)	Specification 2 aOR (95% CI)	Specification 3 aOR (95% CI)
**Urban area**			
Low-SEP	1.48 (0.85–2.58)	1.27 (0.82–1.96)	0.89 (0.61–1.28)
Middle-SEP	1.30 (0.71–2.40)	1.34 (0.87–2.07)	1.00 (0.74–1.36)
High-SEP	Ref	Ref	Ref
**Rural area**			
Low-SEP	1.46 (0.48–4.42)	2.40**(1.06–5.41)	0.70 (0.42–1.17)
Middle-SEP	0.67 (0.25–1.83)	1.27 (0.64–2.50)	0.63**(0.41–0.96)
High-SEP	Ref	Ref	Ref

Footnote: ** *p* < 0.05, Ref_ reference group. All models were adjusted for sex, age-group, marital status, education level, presence of chronic conditions and functional status. **Specification 1**: Did not receive healthcare despite reporting a healthcare need within the past three years, **Specification 2**: Did not receive healthcare or received care from a non-medical facility, and **Specification 3**: Did not receive healthcare or received care from a non-medical facility or private pharmacy

### Predicted probability of unmet healthcare needs across three outcome specifications

**Fig. 2:**
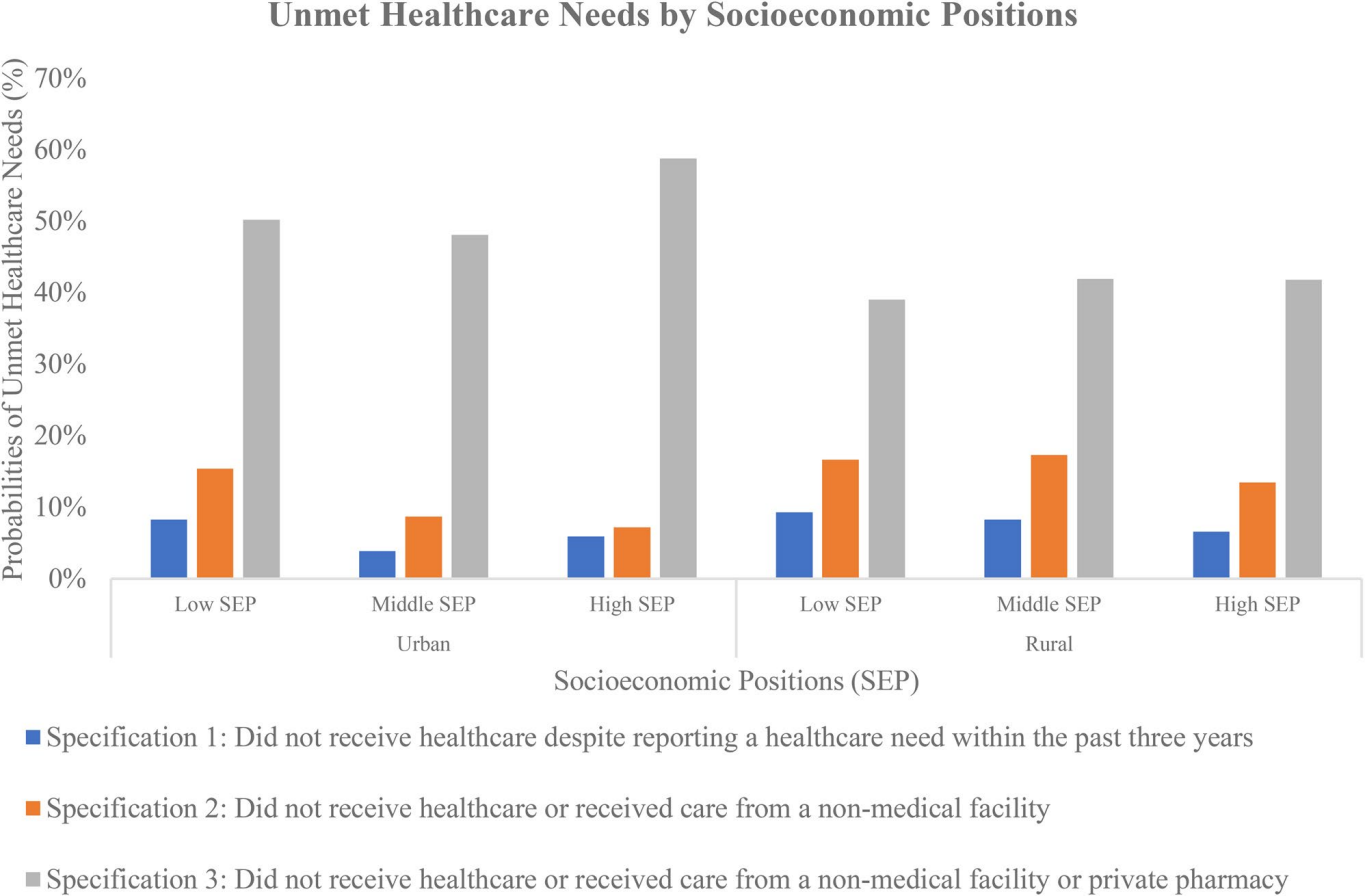
Predicted probability of unmet healthcare needs by socioeconomic position and residential area across three alternative specifications, WHS+ Cambodia 2023

Figure 2 illustrates the predicted probability of unmet healthcare needs across the three outcome specifications stratified by SEP and residential area. Overall, the predicted probability of unmet healthcare needs increased substantially from Specification 1 to Specification 3 in both urban and rural areas, reflecting the broader classification of unmet healthcare need when non-medical providers and private pharmacies were included.

In urban areas, differences across socioeconomic groups were relatively small under Specification 1 and Specification 2, although low-SEP individuals generally showed higher predicted probabilities than middle- and high-SEP groups. Under Specification 3, predicted probabilities increased markedly across all SEP groups, and differences between socioeconomic groups became less distinct.

In rural areas, a similar upward trend across specifications was observed. Under Specification 2, low- and middle-SEP groups showed comparatively higher predicted probabilities of unmet healthcare needs than the high-SEP group, consistent with the regression findings. However, under Specification 3, predicted probabilities increased across all SEP groups and converged to similar levels.

## Discussion

Access to timely, affordable, and quality healthcare is central to achieving health equity and universal health coverage (UHC) [40]. In Cambodia, as in many LMICs, structural, financial, and geographic barriers continue to constrain access to formal healthcare services [41]. Using nationally representative data and three alternative operational definitions of unmet healthcare need, this study shows that both the estimated prevalence of unmet healthcare needs and the socioeconomic and geographic patterns vary across different definitions of healthcare access. To our knowledge, this is the first study in Cambodia to examine these inequalities using multiple provider-based definitions, thereby highlighting how measurement choices can shape the identification of equity gaps and policy priorities.

### Socioeconomic and geographic inequalities in access to formal healthcare

Our findings showed that the prevalence and socioeconomic patterning of unmet healthcare needs differed substantially across specifications and residential areas. Under Specification 1, which captures self-reported non-receipt of care despite healthcare need, no statistically significant socioeconomic gradient was observed in either urban or rural populations after adjustment. However, unmet healthcare need increased substantially when broader provider-based definitions were applied. Under Specification 2, which classified reliance on non-medical facilities as unmet healthcare need, socioeconomic inequalities became more apparent, particularly among rural populations. Rural residents in the low-SEP group had significantly higher odds of unmet healthcare need compared with high-SEP rural residents. These findings suggested that inequalities were more pronounced when unmet healthcare need was defined in relation to access to formal healthcare facilities, while broader definitions of unmet healthcare need that included pharmacy-based care revealed different patterns across SEP and residential area. These findings are consistent with previous studies in China and other settings, showing that rural residents face persistent barriers to healthcare access, including limited service availability, limited healthcare workforce distribution, and transportation constraints [41–44]. The stronger association observed in rural areas is consistent with evidence from Cambodia [45] and other LMICs, [46–48], which consistently demonstrate that financial hardship is a primary barrier to accessing formal healthcare. A prior qualitative study in Cambodia further supports these findings, highlighting high out-of-pocket costs, workforce shortages, transportation challenges, and limited rural service availability as major obstacles to healthcare access [41]. Together, these findings suggest that lower socioeconomic position in rural settings remains closely linked to dependence on non-medical facilities and constrained access to qualified care.

### Pharmacy use, care pathways, and contextual differences

When private pharmacies were additionally included in the definition of unmet healthcare need (Specification 3), the pattern of association changed considerably. The prevalence of unmet healthcare need increased markedly across all socioeconomic groups, reaching over 40% nationally. Predicted probabilities similarly increased in both urban and rural populations, with a more pronounced increase observed in urban areas. In urban populations, socioeconomic differences became less distinct. In contrast, rural areas continued to show some socioeconomic variation, with middle-SEP individuals having significantly lower odds of unmet healthcare needs compared with high-SEP rural residents. This finding reflects the central role of pharmacies within Cambodia’s pluralistic healthcare system. Pharmacies are widely available and frequently serve as accessible first points of contact, particularly in urban areas [49, 50]. Their use may reflect convenience, shorter waiting times, and easier access rather than complete exclusion from healthcare services. At the same time, the utilization pattern observed in rural areas suggests that pharmacy use and its relationship with unmet healthcare needs may vary according to local healthcare availability, affordability, and care-seeking pathways. Similar patterns have been reported in Vietnam [51] and India [52], where pharmacies are commonly used as convenient alternatives to formal medical consultation. However, this differs from findings in Bangladesh, where pharmacy use is more common among lower-income populations, [53] highlighting the context-specific nature of care-seeking pathways. The inclusion of private pharmacies in Specification 3 therefore captures a broader dimension of healthcare access, distinguishing between formal clinical care and alternative care pathways. While pharmacies may improve access to medicines and provide a practical entry point into healthcare systems, they do not necessarily ensure appropriate diagnosis, continuity of care, or clinical management [52, 56]. Consequently, whether pharmacy-based care should be considered adequate healthcare access remain conceptually and policy relevant. Together, these findings demonstrate that unmet healthcare need is a multidimensional construct whose interpretation depends substantially on how healthcare access is defined. Measurement approaches that distinguish between provider types therefore provide important additional insight into healthcare inequalities and care pathways.

### Interpretation variation across specifications

The variation in findings across specifications suggests that unmet healthcare need is not solely determined by structural access barriers but shaped by healthcare-seeking behaviors and expectations regarding care. This interpretation is supported by prior research showing that self-reported unmet healthcare need is influenced by expectations, health literacy, and prior healthcare experiences [55]. From a health-seeking behaviors perspective, individuals with high SEP may have greater expectations regarding service quality, convenience, and responsiveness, and may therefore report unmet needs even when some form of care (e.g., pharmacy use) is accessed. In contrast, individuals with lower SEP or those in rural areas may normalize structural barriers and underreport unmet need despite facing greater access constraints. This perspective helps explain why socioeconomic differences appeared limited under Specification 1 yet became more pronounced under Specification 2. Self-reported receipt care may obscure inequalities in access to qualified providers if individuals rely on informal or non-medical care pathways. Conversely, broader definitions that distinguish provider types may better capture inequalities in access to formal healthcare services.

The convergence of predicted probabilities under Specification 3 further suggests that pharmacy use cuts across socioeconomic groups, although the reasons for such use may differ. Among urban or higher-SEP populations, pharmacy use may reflect convenience and preference, whereas among rural or lower-SEP populations it may reflect limited alternatives or barriers to formal care. This finding is consistent with broader literature from LMICs, where perceived need and acceptability are closely linked with personal preferences or circumstances, strongly influencing self-reported unmet healthcare needs [56]. Previous studies in Cambodia highlighted that formal healthcare services may be viewed as time-consuming, costly, or administratively complex, prompting individuals to seek conveniently accessible alternatives such as pharmacies [50]. These perceptions help explain why reported unmet healthcare needs may not always align with objective barriers to access.

High levels of reported satisfaction with healthcare in LMICs, despite well-documented limitations, further complicate interpretation [57]. Patient satisfaction does not necessarily reflect technical quality of care, particularly where expectations are modest or where accessible alternatives are limited. In Cambodia and similar settings, reliance on private pharmacies and non-medical facilities may offer convenience and partial safety net but not always ensuring appropriate diagnosis or treatment [50, 52, 54].

### Implications for monitoring and policy

These findings have important implications for both monitoring and policy. Reliance on a single self-reported measure of unmet healthcare need may overlook inequalities related to provider type and quality of access. Monitoring systems should therefore incorporate complementary measures that distinguish between lack of access to formal care, reliance on non-medical facilities, and pharmacy-based care. Policy responses should also recognize the differing patterns observed across residential settings. In rural areas, where socioeconomic inequalities were most evident under Specification 2, efforts should prioritize strengthening primary healthcare infrastructure, improving transportation and referral systems, expanding outreach services, and addressing healthcare workforce shortages through rural deployment and retention strategies. At the same time, the widespread reliance on pharmacies and non-medical facilities highlights the need for pragmatic engagement with pluralistic healthcare systems. Rather than treating these providers solely as substitutes for formal care, policy approaches should consider stronger regulation, referral training, and integration mechanisms to improve quality and continuity of care. Across all populations, improving health literacy, community engagement, and trust in formal healthcare services remain essential to support appropriate care-seeking and reduce avoidable unmet healthcare needs.

## Strengths and limitations

This study used data from the 2023 WHS+, a large, nationally representative sample of Cambodian adults, supporting the generalizability of the findings. The availability of detailed information on healthcare utilization across public and private facilities, as well as non-medical facilities, allowed for a comprehensive assessment of care-seeking patterns. In addition, applying three alternative specifications of unmet healthcare needs enabled a more nuanced examination of socioeconomic and geographic inequalities in healthcare access within a lower-middle-income country context. In addition, stratifying the analysis by residential area provides insight into how socioeconomic gradients in healthcare access differ between urban and rural populations. The analytical approach distinguished confounders from potential mediators, thereby reducing the risk of overadjustment.

Several limitations should be acknowledged. First, reliance on self-reported data may have introduced recall bias, social desirability bias, and measurement error in reporting healthcare need and utilization. The three-year recall period may have further contributed to misclassification, as participants may not accurately remember past healthcare experiences; however, this recall period was inherent to the survey design and could not be modified by the authors. In addition, healthcare utilization was assessed using questions referring to both the most recent healthcare need and healthcare use within the preceding three years. This approach may not fully capture usual care-seeking patterns, particularly in settings where individuals alternate between formal and informal providers depending on symptoms, cost, or availability.

Second, the study did not assess the quality or clinical appropriateness of care received from non-medical facilities or pharmacies, limiting the interpretation of healthcare adequacy. Relatedly, the different operational definitions of unmet healthcare need may have influenced the observed inequality patterns, highlighting that the results are sensitive to measurement choices. Third, individuals seeking care abroad wereexcluded because their healthcare experiences were not comparable to the domestic healthcare system; however, they represented a very small proportion of the sample (0.67%), and the overall impact on the findings is likely to be minimal.

Fourth, the analyses did not adjust for health insurance status, smoking, or alcohol use. These factors were considered likely mediators rather than confounders, but their exclusion may still have resulted in residual confounding if they were associated with both SEP and unmet healthcare needs through alternative pathways. Available information on health insurance was also limited, with only 17.1% of participants reporting coverage and insufficient detail to distinguish between different types of schemes or levels of financial protection. Including this variable without adequate specification could have introduced misclassification and biased interpretation.

Finally, supply-side factors such as healthcare facility availability, geographic distribution, and workforce density were not captured, limiting the ability to contextualize observed inequalities within the health system structure. The cross-sectional design also limits assessment of the direction or timing of the relationship between SEP and unmet healthcare needs. Generalizability may be limited to settings with similar health system organization, socioeconomic structures, and patterns of healthcare access.

## Conclusion

Unmet healthcare needs in Cambodia varied substantially depending on how access was defined, revealing distinct socioeconomic and geographic inequalities. These findings highlight that estimates unmet healthcare need are sensitive to measurement approaches and may capture different dimensions of access and care-seeking behavior. From a policy perspective, improving equity will require strengthening rural health services through better infrastructure, workforce distribution, and referral systems. The results underscore the importance of policy approaches that go beyond financial protection alone and consider geographic barriers, service availability, and quality of care. Attention to the role of private and non-medical facilities is also essential in efforts to advance progress towards universal health coverage. In addition, monitoring systems should use multiple definitions of unmet healthcare and need to better identify inequities and inform targeted interventions.

## Supplementary Information

Below is the link to the electronic supplementary material.

**Supplementary table S1:** Frequency and weighted proportion of participants reporting their healthcare access and the distribution of unmet healthcare needs across three specifications among Cambodian adults, WHS+ Cambodia 2023 Variables included in PCA for rural and urban households Adjusted asociations between socioeconomic position and unmet healthcare needs among urban Cambodian adults, WHS+ Cambodia 2023 Adjusted asociations between socioeconomic position and unmet healthcare needs among rural Cambodian adults, WHS+ Cambodia 2023

## Data Availability

The data analysed in this study are part of theWHS+ and are intended to be made publicly available through the World Health Organization’s survey data repository (https://apps.who.int/healthinfo/systems/surveydata/index.php/collections/central). At the time of submission, the Cambodia WHS+ 2023 dataset is not yet publicly accessible. Access to the data may be requested from the World Health Organization and the National Institute of Public Health, Cambodia, subject to relevant approvals.
