# Correction: Socioeconomic and geographic inequalities in unmet healthcare needs in Cambodia: evidence from a national cross-sectional study

**DOI:** 10.1186/s12939-026-02961-5

**Published:** 2026-07-29

**Authors:** Khin Thiri Maung, Monica Hunsberger, Heng Sopheab, Nawi Ng, Ailiana Santosa

**Affiliations:** 1 School of Public Health and Community Medicine, Institute of Medicine, Sahlgrenska Academy, University of Gothenburg, Gothenburg, Sweden; 2 School of Public Health, National Institute of Public Health, Phnom Penh, Cambodia

**Correction: Int J Equity Health (2026) 25:156**

10.1186/s12939-026-02922-y

Following the publication of the original article [1], it was noted that in the sentence beginning ‘In addition, participants were excluded if they reported seeking healthcare abroad…’ the value ‘(ADL) (n = 16, 3.2%)’ should have read ‘(ADL) (n = 16, 0.32%)’.

The original article [1] has been corrected.
