# Supplementary material for: Socioeconomic and geographic inequalities in unmet healthcare needs in Cambodia: evidence from a national cross-sectional study
Source: Int J Equity Health. 2026 Jun 18;25:156. doi: 10.1186/s12939-026-02922-y (PMC13285095; doi:10.1186/s12939-026-02922-y)
Supplement: Supplementary file 1 — Supplementary table S1: Frequency and weighted proportion of participants reporting their healthcare access and the distribution of unmet healthcare needs across three specifications among Cambodian adults, WHS+ Cambodia 2023 Variables included in PCA for rural and urban households Adjusted asociations between socioeconomic position and unmet healthcare needs among urban Cambodian adults, WHS+ Cambodia 2023 Adjusted asociations between socioeconomic position and unmet healthcare needs among rural Cambodian adults, WHS+ Cambodia 2023 [file 12939_2026_2922_MOESM1_ESM.docx]

**APPENDIX**

**Supplementary Table S1: Frequency and weighted proportion of participants reporting their healthcare access and the distribution of unmet healthcare needs across three specifications in Cambodia, World Health Survey Plus (WHS+) 2023**

| **CHARACTERISTICS** | **n** | **Weighted %** | **Specification 1** | **Specification 2** | **Specification 3** |
| --- | --- | --- | --- | --- | --- |
| Those who needed healthcare | 5001 | 94.80 |  |  |  |
| Those who had self-reported met need among participants who needed healthcare | 4623 | 92.4 |  |  |  |
| Those who had self-reported unmet need among participants who needed healthcare | 378 | 7.6 |  |  |  |
| Sources of healthcare most frequently used by participants with self-reported met needs |  |  |  |  |  |
| National Hospital | 88 | 1.9 |  |  |  |
| Referral Hospital | 200 | 4.3 |  |  |  |
| Health center/Health Post/ Provincial | 364 | 7.9 |  |  |  |
| Others (Public) | 5 | 0.1 |  |  |  |
| Private Hospital | 470 | 10.2 |  |  |  |
| Private Clinics | 1219 | 26.4 |  |  |  |
| Home/Trainer health worker/ Nurse | 479 | 10.4 |  |  |  |
| Other (Private) | 38 | 0.8 |  |  |  |
| Private Pharmacy | 1406 | 30.4 |  |  |  |
| Shop selling drugs | 112 | 2.4 |  |  |  |
| Magician | 15 | 0.3 |  |  |  |
| Other (Non-medical) | 227 | 4.9 |  |  |  |
| Number (n) |  |  | 378 | 732 | 2138 |
| Percentage (%) |  |  | 7.6 | 14.6 | 42.8 |

Grey highlights show variables included under the three unmet healthcare needs specifications.

Footnote: n= frequency, % = weighted row percentage**. Specification 1:** Did not received healthcare despite reporting a healthcare need within the past three years, **Specification 2:** Did not receive healthcare or received care from a non-medical facility, and **Specification 3:** Did not receive healthcare or received care from a non-medical facility or private pharmacy.

**Supplementary Table S2: Variables Included in** PCA **for Rural and Urban Households**

| **Variables** | **Rural Households** | **Urban Households** |
| --- | --- | --- |
| Type of wall materials | ✓ | — |
| Source of drinking water | ✓ | — |
| Television | ✓ | ✓ |
| Motorcycle | ✓ | ✓ |
| Car or Truck | ✓ | ✓ |
| Electricity access | ✓ | — |
| Bicycle | ✓ | ✓ |
| Washing machine | ✓ | ✓ |
| Refrigerator | ✓ | ✓ |
| Tractor | ✓ | — |
| Radio | ✓ | ✓ |
| Computer | — | ✓ |
| Internet access | — | ✓ |
| Livestock | ✓ | — |
| Own any agricultural land | ✓ | ✓ |
| Own dwelling other than where you live | ✓ | ✓ |

Footnote: ✓ = included in PCA, — = not included in PCA

| **Characteristics** | **Specification 1** | **Specification 2** | **Specification 3** |
| --- | --- | --- | --- |
| **Socioeconomic Status** |  |  |  |
| Low | 1.48(0.85 - 2.58) | 1.27(0.82 - 1.96) | 0.89(0.61 - 1.28) |
| Middle | 1.30(0.71 - 2.40) | 1.34(0.87 - 2.07) | 1.00(0.74 - 1.36) |
| High | Ref | Ref | Ref |
| **Age group (in year)** |  |  |  |
| 18-49 | Ref | Ref | Ref |
| 50-59 | 1.47(0.90 - 2.39) | 1.36(0.84 - 2.21) | 1.05(0.71 - 1.54) |
| 60+ | 0.29***(0.15 - 0.57) | 0.66*(0.40 - 1.08) | 0.60***(0.42 - 0.87) |
| **Sex** |  |  |  |
| Men | Ref | Ref | Ref |
| Women | 0.67* (0.42 - 1.08) | 0.79 (0.53 - 1.18) | 0.53***(0.40 - 0.70) |
| **Marital status** |  |  |  |
| Currently Married | Ref | Ref | Ref |
| Divorced/Widowed/Never Married | 0.50 (0.22 - 1.15) | 0.87 (0.55 - 1.40) | 0.99 (0.72 - 1.35) |
| **Education level** |  |  |  |
| No education & Incomplete primary | 0.97(0.48 - 1.97) | 0.87(0.55 - 1.40) | 1.45**(1.03 - 2.03) |
| Completed primary | 0.71(0.38 - 1.32) | 1.21(0.72 - 2.04) | 1.07(0.72 - 1.59) |
| Completed secondary and above | Ref | Ref | Ref |
| **Presence of chronic conditions** |  |  |  |
| None | Ref | Ref | Ref |
| One | 0.55(0.26 - 1.16) | 0.86(0.53 - 1.38) | 0.82(0.55 - 1.22) |
| Two or more | 1.23(0.57 - 2.63) | 0.71(0.41 - 1.22) | 0.70 (0.46 - 1.06) |
| **Functional status** |  |  |  |
| No difficulty | Ref | Ref | Ref |
| With difficulty | 1.00 (0.57 - 2.63) | 0.80 (0.47 - 1.36) | 1.32**(1.00 - 1.75) |

**Supplementary table S3: Adjusted asociations between Socioeconomic Position and Unmet Healthcare Needs among Urban Cambodian Adults, WHS+ Cambodia 2023**

Footnote: ** p<0.05, Ref_ reference group. All models were adjusted for sex, age-group, marital status, education level, presence of chronic conditions and functional status. **Specification 1:** Did not received healthcare despite reporting a healthcare need within the past three years, **Specification 2:** Did not receive healthcare or received care from a non-medical facility, and **Specification 3:** Did not receive healthcare or received care from a non-medical facility or private pharmacy.

**Supplementary table S4: Adjusted asociations between Socioeconomic Position and Unmet Healthcare Needs among Rural Cambodian Adults, WHS+ Cambodia 2023**

| **Characteristics** | **Specification 1** | **Specification 2** | **Specification 3** |
| --- | --- | --- | --- |
| **Socioeconomic Status** |  |  |  |
| Low | 1.46(0.48 - 4.42) | 2.40**(1.06 - 5.41) | 0.70(0.42 - 1.17) |
| Middle | 0.67(0.25 - 1.83) | 1.27(0.64 - 2.50) | 0.63**(0.41 - 0.96) |
| High | Ref | Ref | Ref |
| **Age group (in year)** |  |  |  |
| 18-49 | Ref | Ref | Ref |
| 50-59 | 0.52**(0.28 - 0.97) | 0.99(0.60 - 1.63) | 0.96(0.63 - 1.46) |
| 60+ | 0.76(0.19 - 2.96) | 1.15(0.50 - 2.61) | 1.01(0.58 - 1.75) |
| **Sex** |  |  |  |
| Men | Ref | Ref | Ref |
| Women | 1.62* (0.92 - 2.84) | 1.49 (0.98 - 2.25) | 0.93 (0.64 - 1.35) |
| **Marital status** |  |  |  |
| Currently Married | Ref | Ref | Ref |
| Divorced/Widowed/Never Married | 1.40 (0.70 - 2.80) | 1.22 (0.63 - 2.38) | 1.46 (0.93 - 2.29) |
| **Education level** |  |  |  |
| No education & Incomplete primary | 0.93(0.44 - 1.98) | 0.69(0.32 - 1.47) | 1.06(0.75 - 1.49) |
| Completed primary | 0.49*(0.22 - 1.10) | 0.69(0.32 - 1.48) | 1.56**(1.02 - 2.38) |
| Completed secondary and above | Ref | Ref | Ref |
| **Presence of chronic conditions** |  |  |  |
| None | Ref | Ref | Ref |
| One | 1.74(0.84 - 3.63) | 1.38(0.74 - 2.57) | 1.09(0.74 - 1.61) |
| Two or more | 0.69(0.18 - 2.74) | 0.85(0.22 - 3.31) | 1.02(0.57 - 1.82) |
| **Functional status** |  |  |  |
| No difficulty | Ref | Ref | Ref |
| With difficulty | 0.75 (0.31 – 1.83) | 0.95 (0.45 - 2.02) | 0.81 (0.54 - 1.21) |

Footnote: ** p<0.05, Ref_ reference group. All models were adjusted for sex, age-group, marital status, education level, presence of chronic conditions and functional status. **Specification 1:** Did not received healthcare despite reporting a healthcare need within the past three years, **Specification 2:** Did not receive healthcare or received care from a non-medical facility, and **Specification 3:** Did not receive healthcare or received care from a non-medical facility or private pharmacy.
